# Promising impacts of *Achillea* spp., beyond A medicinal plant, against toxins, toxicities, and injuries: *In vivo* and *in vitro* mechanisms

**DOI:** 10.1016/j.bbrep.2025.102023

**Published:** 2025-04-22

**Authors:** Mohammad Mahdi Dabbaghi, Mohammad Saleh Fadaei, Maral Goldoozian, Mohammad Reza Fadaei, Vafa Baradaran Rahimi, Vahid Reza Askari

**Affiliations:** a Clinical Research Development Unit, Imam Reza Hospital, Faculty of Medicine, Mashhad University of Medical Sciences, Mashhad, Iran; b Department of Pharmaceutics, School of Pharmacy, Mashhad University of Medical Sciences, Mashhad, Iran; c Department of Cardiovascular Diseases, Faculty of Medicine, Mashhad University of Medical Sciences, Mashhad, Iran

**Keywords:** *Achillea*, *Achillea* spp., Natural toxin, Chemical toxin, Toxicity

## Abstract

Natural toxins produced by various living organisms pose significant risks to health, food security, and environmental balance through inhalation, ingestion, and other exposure routes. This review focuses on the ameliorative effects of different *Achillea* species, which comprise over 130 perennial herbs known for their therapeutic properties. A systematic examination of data from Scopus, PubMed, and Web of Science was conducted, encompassing various studies without date restrictions, ensuring a comprehensive selection of articles based on full-text availability. The results of this study indicate that *Achillea millefolium* exhibits anti-hyperglycemic and anti-hyperlipidemic properties, enhances collagen proliferation regulation, suppresses inflammatory responses, and displays significant antioxidant activity. Similarly, *A. wilhelmsii* has been shown to have hepatoprotective effects, as evidenced by reduced malondialdehyde levels and increased total thiol concentrations. *A. fragrantissima* has also been demonstrated to have cardioprotective effects, with a decrease in inflammatory markers and edema levels. The protective benefits of other species within the *Achillea* genus extend to various toxins. This comprehensive review underscores the potential of *Achillea* species as natural remedies for combating toxicities and promoting health.

## Highlights

• *Achillea* Spp. attenuates oxidative stress and inflammation.
• *Achillea* Spp. lessens apoptosis, and oxidative DNA damage and prevents tumorigenesis.
• Pretreatment with *Achillea* Spp. greatly reduces the oxidative stress brought on by radiation.
• *Achillea* Spp. detoxifies microbial toxins.
• *Achillea* Spp. ameliorates the adverse effects of drugs and chemical toxins.

## Graphical abstract

**Figure:**
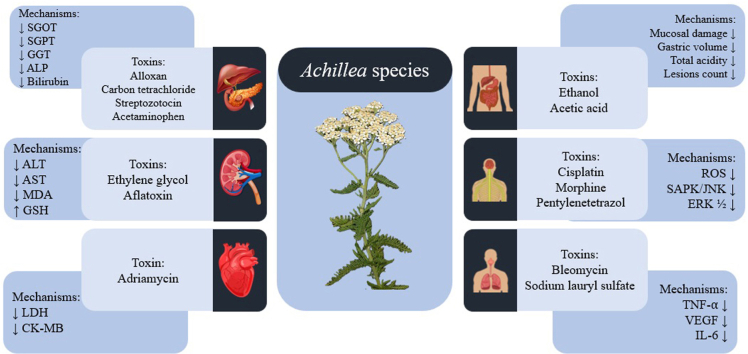

## Abbreviations

ALP: Alkaline Phosphatase
ALT: Alanine Transaminase
AM-EO: *Achillea millefolium* Essential Oil
ATP: Adenosine Triphosphate
AST: Aspartate Aminotransferase
BAF: Bioaccumulation factors
Bax: Bcl-2-associated X protein
BCF: Bioconcentration Factors
Bcl-2: B cell lymphoma 2
BUN: Blood Urea Nitrogen
CAT: Catalase
COM: Calcium Oxalate Monohydrate
COX-2: Cyclooxygenase-2
DM: Diabetes Mellitus
DM2: Diabetes Mellitus Type 2
EAE: Ethyl Acetate Extract
FBG: Fasting Blood Glucose
GIS: Gastrointestinal System
GOT: Glutamic-Oxaloacetic Transaminase
GPT: Glutamic Pyruvic Transaminase
GPx: Glutathione Peroxidase
GSH: Glutathione
GST: Glutathione S-Transferase
HDL: High Density Lipoprotein
HO-1: Heme Oxygenase-1
IL: Interleukin
iNOS: inducible Nitric Oxide Synthase
JNK: Jun N-terminal Kinase
LDL: Low-Density Lipoprotein
LPO: Lipid Peroxide
LPS: Lipopolysaccharide
MDA: Malondialdehyde
MPO: Myeloperoxidase
NF-κb: Nuclear Factor Kappa Light Chain Enhancer of Activated B Cells
NO: Nitric Oxide
NAPQI: N-Acetyl-P-Benzoquinone Imine
PTZ: Pentylenetetrazole
ROS: Reactive Oxygen Species
SGOT: Serum Glutamic-Oxaloacetic Transaminase
SGPT: Serum Glutamic Pyruvic Transaminase
SLS: Sodium Lauryl Sulfate
SOD: Superoxide Dismutases
STZ: Streptozotocin
TG: Triglyceride
TGF-β: Transforming Growth Factor Beta
TLR-4: Toll-like receptor 4
TNF-α: Tumor Necrosis Factor-alpha
VLDL: Very Low-Density Lipoprotein

## 1 Introduction

The *Achillea* genus belongs to the Asteraceae family and includes over 130 perennial herb species native to the northern hemisphere, especially from Europe to Asia. It prefers almost dry or semi-dry regions with moderate climates [1]. Asteraceae is the most prominent vascular plant family and can be found all over the world, but they are most common in arid and semi-arid regions of subtropical and lower temperate latitudes. *Achillea* is represented in Iran by 19 species, 7 of which are classified as endemic, and in Turkey by, 46 taxa, 25 of which are endemic [2,3]. For more than 3000 years, *Achillea millefolium*, as one of the most famous and widespread species of *Achillea* genus, has been listed as one of the most used plant species in traditional medicine [4]. *A. millefolium* is distributed throughout Europe, Western Asia, and North America [5]. It often grows in open woodlands and grasslands. The plant usually flowers from May to June, and its growth is more active in the spring [2].

The plant is a perennial with a slender rootstock that produces numerous roots and runners. It grows up to 50 cm tall with a blunt, succulent scale at each node. The leaves are 5–20 cm long and are arranged in a spiral pattern near the center and end of the stem. Its leaves are almost feathery and hairy and are bipinnate or tripinnate. Flowers are usually white but may be pink or light purple, with flattened heads at the ends of stems and branches. The petals are densely arranged in flattened clusters. The fruits are elongated achenes, 2 mm in size, with winged margins [6].

*Achillea fragrantissima* is a desert plant that has been studied for various pharmacological effects. *A. fragrantissima* is a plant native to countries in northeastern Africa and the Middle East [7]. This plant can grow 30–60 cm tall and has small yellow flowers [8]. In traditional medicine, this plant has been used for gastrointestinal disorders, stomach pain, and hepatobiliary diseases [9]. *A. fragrantissima* has been shown to have anti-inflammatory and antioxidant effects that can be used in various diseases such as neuroinflammatory diseases, malignancies, and diabetes mellitus (DM) [8]. Another species of this genus is *Achillea santolina*. *A. santolina* is a plant native to warm regions such as Europe and Asia. It is traditionally used for digestive problems, cramps, fever, and wounds [10]. Various studies have investigated the pharmacological effects of *A. santolina*, such as antimicrobial, anti-inflammatory, antidiabetic, cardiovascular, and antioxidant effects [11]. *Achillea biebersteinii* is another plant of the *Achillea* genus. Its height is 30–60 cm, and it has simple or branched stems with leaves that are 10 cm long [12]. In traditional remedies, this plant is used for stomach pain and wounds [13]. Its antioxidant and anti-inflammatory effects have been studied. Various studies have also demonstrated the antifungal and antibacterial properties of its extract [14]. Another plant of this genus that may have protective effects against toxins is *Achillea odorata*. This plant is found in the Mediterranean region, North America, various parts of Europe, western and eastern Asia, Australia, and New Zealand [15]. In traditional medicine, it has been used as an anti-diabetic, allergic rhinitis, anti-rheumatic, and anti-inflammatory. Its essential oil has been reported to have antimicrobial and anti-inflammatory effects [16]. In addition, *A. wilhelmsii* has been used in traditional remedies for centuries. The aerial parts of *A. wilhelmsii* are used for therapeutic conditions such as lung diseases [17]. It has also been used for hypertension, hyperlipidemia, inflammation, spasm, and uterine contraction [18].

**Fig. 1:**
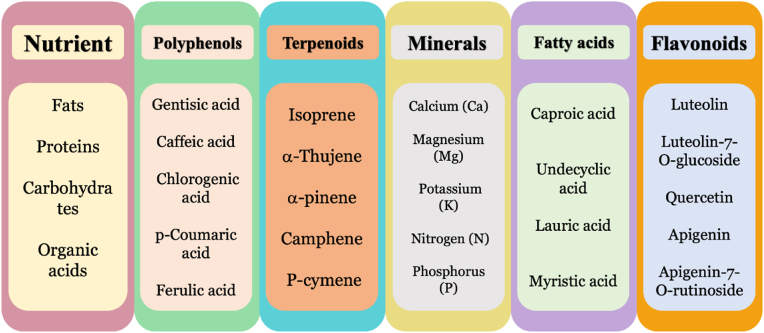
Active constituents of *Achillea* species (Data extracted from Refs. [[31], [32], [33], [34], [35]]).

Toxins are biomolecules produced primarily by bacteria, fungi, insects, plants, vertebrates, and invertebrates for defense purposes. When inhaled, injected, ingested, or absorbed, these molecules cause damage to other organisms [19]. The effects of toxins are often permanent, resulting in lifelong health damage. Toxins have a significant impact on health, food safety, and environmental safety [[20], [21], [22], [23], [24], [25]]. *Achillea* essential oils and their extracts showed a wide range of properties such as antioxidant, antibacterial, antifungal, antimicrobial, and herbicidal activities. *A. millefolium* has been used for centuries in traditional medicine as an appetite stimulant, herbal teas, lotions and ointments for various conditions such as skin inflammation, wounds, headache and abdominal discomfort [[26], [27], [28], [29], [30]]. Some active constituents such as polyphenols, terpenoids, and flavonoids are found in *Achillea* species, which are shown in Fig. 1 [[31], [32], [33], [34], [35]].

**Fig. 2:**
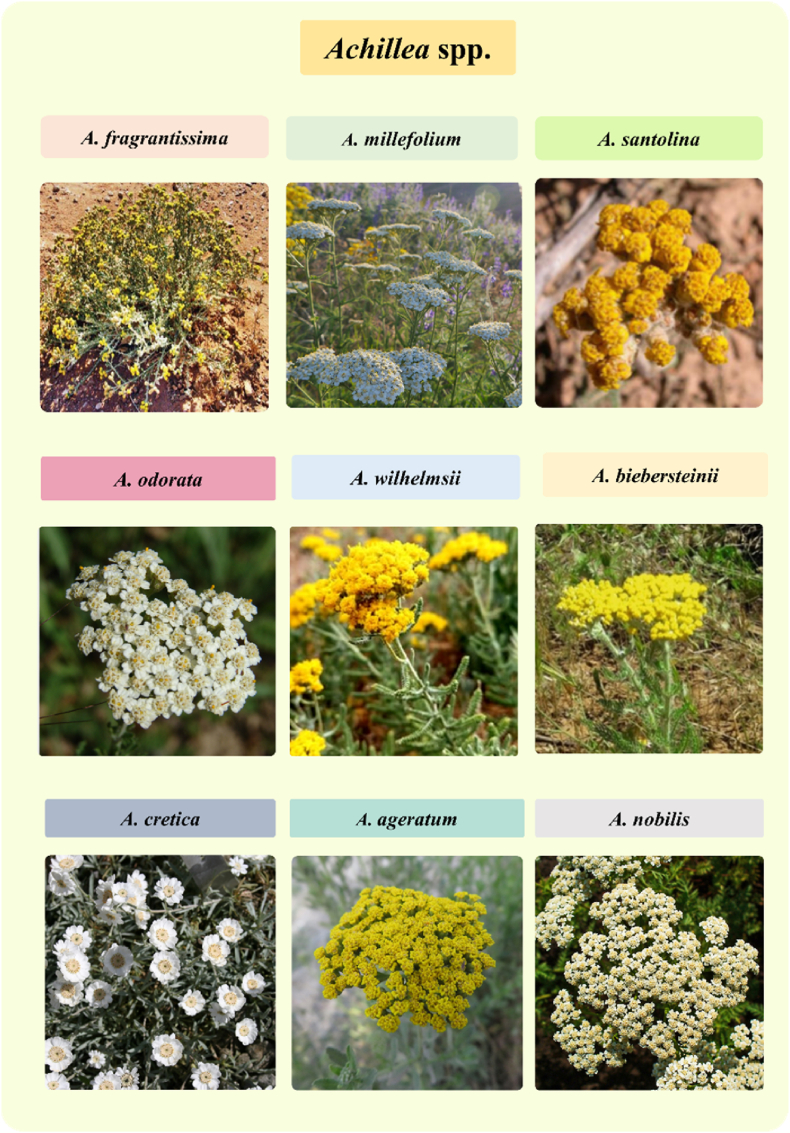
Picture of some important *Achillea* species [[174], [175], [176], [177], [178], [179]].

Compounds like Quercetin and Apigenin, contribute to anti-inflammatory effects by modulating pathways such as nuclear factor kappa light chain enhancer of activated B cells (NF-κb) and inhibiting cyclooxygenase-2 (COX-2). The antioxidant properties help protect against oxidative stress and inflammation, which are beneficial in wound healing and other condition [36,37]. We live in a world where our health is affected by a variety of factors such as climate, food, and toxins through multiple administrative roots. Toxic agents can cause organ failure due to their physical, chemical, or biological properties. Independent characteristics such as age, diet, disease, pregnancy, and other relevant factors determine the severity of damage [[21], [22], [23], [24], [25]]. Throughout history, people have extensively used natural substances derived from plants, which were researched, isolated, and transformed into modern medicine to prevent or treat various diseases [38]. Various diseases and inflammatory reactions associated with various toxins could be hindered and controlled with the help of various species of *Achillea*. For example, *A. millefolium* was found to have an ameliorative effect against alloxan [39], carbon tetrachloride (CCl_4_) [40], bleomycin [41], cisplatin [42], ethanol [43], ethylene glycol [44], lipopolysaccharide (LPS) [45], morphine [46], sodium lauryl sulfate (SLS) [47], and streptozotocin (STZ) [48], while *A. wilhelmsii*, *A. fragrantissima*, *A. santolina*, *A. biebersteinii*, *A. odorata*, and other species have the same effect against acetaminophen [49], acetic acid [50], pentylenetetrazol (PTZ) [51], adriamycin (Adr) [52], carrageenan [53], and aflatoxin [54]. In the current paper, we discussed more about these important *Achillea* species (Fig. 2) and their application in combating toxicities associated with toxins or some drugs.

## 2 Search strategy

A comprehensive search was performed in Scopus, PubMed, and Web of Science databases without date limitation from inception to date, and selection was based on access to the full text of the articles. *In vitro*, *in vivo*, and clinical studies reporting the protective effects of different *Achillea* extracts were considered and included in this review article. The following medical keywords were searched alone or in combination: "*Achillea*" and "inflammation, fibrosis, toxin, toxic, and toxicity".

## 3 Protective effects of *Achillea* spp. against natural and chemical toxins

### 3.1 Achillea millefolium

The *Achillea* genus, with more than a hundred species, showed a broad spectrum of applications in traditional medicine for diseases of different origins, such as gastrointestinal and hepatobiliary disorders, pneumonia, hemorrhage, wound healing, and rheumatic pain. Extracts from *Achillea* spp. have diverse biological activities and are used in various industries. These plants provide antibacterial, antifungal, and astringent benefits in food, pharmaceutical, and cosmetic applications [55]. *A. millefolium,* or "yarrow," is the most famous species of the genus *Achillea*. It has many pharmacological activities and has been used times to treat several diseases, including malaria, jaundice, and especially hepatitis; it is also prescribed for hemorrhoids, kidney stones, headache, bruises, influenza, cough, pneumonia, high blood pressure, fever, menstrual disorders, rheumatoid arthritis, osteoarthritis, gout, hemorrhagic disorders, cystitis, chicken pox, DM, and, indigestion [6]. Among the phytochemicals present in *A. millefolium* include chlorogenic acid, rutin, luteolin-7-glucoside, schaftoside, vicenin-2, luteolin, 3-*O*-caffeoylquinic acid, caffeic acid hexoside, quercetin, ferulic acid, syringic acid, gallic acid, vanillin, trans(3)-hydroxycinnamic acid, sinapic acid, 4-hydroxybenzoic acid, myrcetin, kaempherol, hyperoside, resveratrol, naringin, naringenin, camphor, β-terpine, β-pinene, santolina triene, terpineol, *p*-cymene, 2-nephthalenamine, borneol, terpine-4-ol, α-terpineol, β-caryophylene, α-eudesmol, α-seline, palmitic acid, linoleic acid, linolenic acid, arachidic acid, behenic acid, and lignoceric acid [55]. The protective effects of *A. millefolium* against natural and chemical toxins are shown in Table 1.

**Table 1:** Protective effects of *A. millefolium* against natural and chemical toxins.

Toxin/Noxious	Model	Extract/Essential Oil/Dose/Concentration	Results	References
Acetic acid	Rats	Aqueous extract (100, and 300 mg/kg/7 days)	− Healing the gastric ulcer (p < 0.05)	[72]
			↓ Gastric lesions (p < 0.05)	
Alloxan	Rats	Aqueous and methanolic extract (250, and 500 mg/kg for 2 weeks)	↓ Cholesterol (p < 0.05)	[59]
			↓ TG (p < 0.05)	
			↓ LDL (p < 0.05)	
			↓ VLDL (p < 0.05)	
			↓ SGOT (p < 0.05)	
			↓ SGPT (p < 0.05)	
			↓ ALP (p < 0.05)	
			↑ Body weight (p < 0.05)	
			↑ Glucose tolerance (p < 0.05)	
Carbon tetrachloride	Rats	Ethanolic extract (100, and 200 mg/kg)	↓ GPT (p = unknown)	[65]
			↓ GOT (p = unknown)	
			↓ ALP (p = unknown)	
Carbon tetrachloride	Albino male mice	Extract (100, and 200 mg/kg)	↓ GPT (p ≤ 0.001)	[40]
			↓ GOT (p ≤ 0.0001)	
			↓ ALP (p ≤ 0.05)	
Carbon tetrachloride	Mice	100, 250, and 500 mg/kg	↓ ALT (p ≤ 0.05)	[153]
			↓ AST (p ≤ 0.05)	
			↓ ALP (p ≤ 0.05)	
			↓ Bilirubin (p ≤ 0.05)	
Carbon tetrachloride	One-day-old male broiler	50 and 100 mg/kg	↓ Serum lipids (p = unknown)	[154]
Cisplatin	Rats	Extract (200, and 400 mg/kg for 14 days)	↓ TNF-α (p < 0.001)	[42]
			↓ MDA (p < 0.001)	
			↓ Caspase-3 expression (p < 0.001)	
			↓ IL-1β (p < 0.001)	
			↑ IL-10 (p < 0.001)	
			↑ SOD (p < 0.001)	
Cyclophosphamide	NMRI male rat	Ethanolic extract (75, 150, and 300 mg/kg)	↑ Body weight (p < 0.001)	[155]
			↑ testis weight (p < 0.01)	
			↓ sperm count (p < 0.001)	
Dextran sulfate sodium	Mice	Essential oil (100 mg/kg for 9 days)	↓ TNF-α (p < 0.001)	[156]
			↓ NF-κb (p < 0.01)	
			↓ IL-6 (p < 0.001)	
			↑ IL-10 (p < 0.01)	
			↑ PPAR-γ (p < 0.001)	
			↑ TGF-β (p < 0.001)	
d-galactoseamine, lipopolysaccharide	Mice	Crude extract (150, 300, and 600 mg/kg)	↓ ALT (p < 0.05)	[75]
			↓ AST (p < 0.05)	
			↓ Mortality (p = unknown)	
Ethanol, indomethacin, and acetic acid	Rats	Aqueous extract of aerial parts (0.3, 0.6, and 1.2 g/kg for 90 days)	↓ Mucosal damage (p < 0.05)	[72]
Ethanol, and acetic acid	Rats	Extract (30, 100, and 300 mg/kg for 7 days)	↓ MPO (p < 0.001)	[43]
			↑ GSH (p < 0.05)	
			↑ SOD (p < 0.05)	
Ethylene glycol	Rats	Hydroalcoholic extract (200 and 400 mg/kg for 30 days)	↓ CaOx deposit (p < 0.05)	[44]
			↓ Urinary oxalate concentration (p < 0.05)	
			↑ Urinary citrate concentration (p < 0.05)	
IR	Human lymphocytes	Extract (10, 50, 100, and 200 μg/ml)	↓ Incidence of micronuclei (p < 0.01)	[157]
LPS	RAW 264.7 Macrophages	Essential oil (20, 40, and 80 μg/ml)	↓ NO (p < 0.05)	[45]
			↓ Superoxide anion production (p < 0.05)	
			↓ Lipid peroxidation (p < 0.05)	
			↓ GSH (p < 0.05)	
			↓ iNOS (p < 0.05)	
			↓ COX-2 (p < 0.05)	
			↓ TNF-α (p < 0.05)	
			↓ IL-6 (p < 0.05)	
			↓ HO-1 (p < 0.05)	
			↓ Inflammatory response (p < 0.05)	
			↑ SOD (p < 0.05)	
			↑ CAT (p < 0.05)	
			↑ GPx (p < 0.05)	
LPS	BV-2 microglial cells	Chlorine-containing guaianolide sesquiterpenoids from *A. millefolium* (100 μM)	↓ NO production (p = unknown)	[158]
Morphine	Male Wistar rats	100, 250, and 500 mg/kg/day for 28 days	↓ MDA (p < 0.01)	[46]
			↓ Cleaved Caspase-3 (p < 0.01)	
			↑ SOD (p < 0.05)	
			↑ GPx (p < 0.05)	
			↑ Bcl-2 (p < 0.05)	
Nicotine	Male rats	1.20 g/kg/day in 1 ml	↑ FSH (p < 0.05)	[159]
			↑ LH (p < 0.05)	
Paclitaxel	Male rats	*A. millefolium* extract (200 and 400 mg/kg for 14 days)	↑ SOD activity (p < 0.001)	[160]
			↓ MDA (p < 0.001)	
			↓ TNF-α (p < 0.001)	
			↓ IL-1β (p < 0.001)	
			↓ NFκB (p < 0.001)	
			↓ Caspase-3 (p < 0.001)	
Sodium lauryl sulfate	Human skin	Extract of Aerial parts	↑ Skin hydration (p < 0.05)	[47]
			↓ EI (p < 0.05)	
Streptozotocin	Rats	Extract (100 mg/kg/day for 14 days)	↓ IL-1β gene expression (p < 0.05)	[48]
			↓ iNOS gene expression (p < 0.05)	
			↓ Glucose level (p < 0.05)	
			↑ Insulin level (p < 0.05)	
			↑ Body weight (p < 0.05)	
Streptozotocin	Rats	Hydroalcoholic extract (25 and 100 mg/kg for 28 days)	↓ Blood glucose (p < 0.01)	[161]
			↓ ALT activity (p < 0.001)	
			↓ AST activity (p < 0.001)	
			↓ TC (p < 0.001)	
			↓ TG (p < 0.001)	
			↓ LDL (p < 0.001)	
			↓ HDL (p < 0.01)	
Streptozotocin	Rats	250 mg/kg hydroalcoholic extract	↓ DAM (p < 0.05)	[85]
			↓ DG (p < 0.05)	
			↓ BUN (p < 0.05)	
			↑ SOD (p < 0.05)	
			↑ GPx (p < 0.05)	
			↓ MDA (p < 0.05)	
			↓ Urea (p < 0.05)	
			↓ creatinine (p < 0.05)	
			↓ Blood glucose (p < 0.05)	
			↓ TG (p < 0.05)	
			↓ LDL (p < 0.05)	
			↓ cholesterol (p < 0.05)	
			↑ HDL (p < 0.05)	
			↓ Bax (p < 0.05)	
			↑ Bcl-2 (p < 0.05)	

#### 3.1.1 Alloxan

Alloxan, a hydrophilic and unstable chemical compound, is similar in shape to glucose, which is why it selectively enters and accumulates in the pancreatic β-cell. Its shape similarity allows it to enter the cytosol through the glucose transporter in the plasma membrane of the β-cell. In addition, the thiol group reactivity of alloxan has been linked to another biological effect, the inhibition of glucose-induced insulin secretion by glucokinase inhibition. This inhibition of glucose-induced insulin secretion has been identified as the primary pathophysiological effect of alloxan resulting from its thiol group reactivity. The thiol groups of the glucose-phosphorylating enzyme glucokinase are highly susceptible to oxidation by alloxan. Inhibition of glucokinase results in decreased glucose oxidation and adenosine triphosphate (ATP) production, which further suppresses glucose-induced insulin secretion. In addition, insulin biosynthesis is inhibited by alloxan through the same mechanism. At higher concentrations, alloxan inhibits several cellular functions, including the oxidation of thiol groups of many critical enzymes such as phosphofructokinase, hexokinase, calmodulin-dependent protein kinase, aconitase, and other proteins. Therefore, it is apparent that the reactivity of the thiol groups of glucokinase is responsible for the inhibition of glucose-induced insulin secretion by alloxan [[56], [57], [58]].

Petlevski et al. [39] investigated the antidiabetic effect of *A. millefolium* on alloxan-induced mice. A total of 72 mice were divided into six groups. Two types of *A. millefolium* extract were administered at the dose of 20 mg/kg for seven days. The glucose and fructosamine serum levels were reduced after treatment with *A. millefolium* extract. Its hypoglycemic effects were even better than acarbose, a new drug used in the treatment of type 2 diabetes mellitus (DM2) in mice. The hypoglycemic and hypolipidemic effects of *A. millefolium* were investigated in alloxan-induced rats. Mustafa et al. [59] divided 30 rats into five groups: normal, diabetic control, diabetic treated with extract at the dose of 250 and 500 mg/kg, and reference control. Extract-treated rats had a significant increase in body weight. They also showed a reduction in serum triglyceride (TG), cholesterol, low-density lipoprotein (LDL), and very low-density lipoprotein (VLDL). These results indicated the anti-hyperglycemic and anti-hyperlipidemic effects of the extract of *A. millefolium*.

#### 3.1.2 Carbon tetrachloride

Carbon tetrachloride (CCl_4_) is known as a routine chelating agent in laboratories and the chemical industry. Its use has been associated with liver damage in several studies [60,61]. In the liver, CCl4 has been shown to alter lipid profiles, oxidative stress markers, total protein, high-density lipoprotein (HDL), liver enzymes, inflammatory markers, hepatocytes, and fiber segmentation [62,63]. In rats, chronic exposure to CCl_4_ results in mutagenicity and DNA fragmentation [64]. The study by Al-Ezzy et al. [40] aimed to investigate the hepatoprotective effects of *A. millefolium* methanolic extract on CCl_4_-induced hepatotoxicity in albino male mice. The study found that the methanolic extract of *A. millefolium* had a hepatoprotective effect on the mice, besides reducing the levels of aspartate aminotransferase (GOT), alanine aminotransferase (GPT), and alkaline phosphatase (ALP) in the mice. The study concluded that the methanolic extract of *A. millefolium* has a hepatoprotective effect on CCl_4_-induced hepatotoxicity in mice. The study by Alzomor and Nada investigated the effects of *A. millefolium* plant extract as a hepatoprotective agent on CCl_4_-induced liver toxicity in female rats. The groups treated only with *A. millefolium* extract at doses of 100 and 200 mg showed no histopathologic changes in liver sections that could be exaggerated from normal. Also, the levels of GPT, GOT, and ALP increased with CCl_4_ treatment, but these liver enzyme levels were reduced in the groups treated with *A. millefolium* extract [65].

#### 3.1.3 Bleomycin

Bleomycin, a chemotherapeutic drug, is developed from the bacterium *Streptomyces verticillus* and belongs to the group of glycopeptides [66]. It has some toxicity to healthy organs such as the lungs [67]. Bleomycin treatment has also been associated with the degradation of double-stranded deoxyribonucleic acid (DNA) in the presence of iron and oxygen through the production of reactive nitrogen species (RNS) and reactive oxygen species (ROS) [68]. *A. millefolium* extract could protect the negative effect of bleomycin on rat lungs in a study by Hemmati et al. [41] in which they divided rats into groups of six animals with induction of 7.5 IU/kg bleomycin sulfate and then for treatment of 400, 800 and 1600 mg/kg (oral) once a day, for 2 weeks. The result showed clear alveolar thickening along with the proliferation of fibroblasts and myofibroblasts and collagen production in the interstitial tissue, leading to pulmonary fibrosis, and *A. millefolium* extract could disrupt the rate of myofibroblast and collagen proliferation.

#### 3.1.4 Cisplatin

Cisplatin, being a wide-ranging chemotherapeutic medication, is employed in clinical studies to combat a diverse range of solid tumors. Nonetheless, hematotoxicity, gastrointestinal toxicity, neurotoxicity, and hepatotoxicity are dose-dependent adverse effects of cisplatin that limit its usage. These major side effects frequently harm patients' quality of life. As a result, we must control or limit the drug's dosage or the following unwanted effects [69]. In this context, researchers wanted to look at methods for reducing these negative impacts. Some of the negative consequences of cisplatin treatment include nausea and vomiting, which are activated by dopamine and dopamine-related areas of the brain, such as the chemoreceptor trigger zone. Dopamine transmission is modulated by tyrosine hydroxylase, dopamine transporter, and dopamine D2 receptor [70]. Okkay and colleagues [42] demonstrated the protective effect of *A. millefolium* against cisplatin-induced ocular toxicity in male rats. The plant extract was administered orally at doses of 200 and 400 mg/kg. Treatment with the extract could reduce the levels of malondialdehyde (MDA), interleukin (IL)-1β, tumor necrosis factor-α (TNF-α), NF-κB, and caspase-3. It also increased levels of superoxide dismutase (SOD) and IL-10. IL-10 is an anti-inflammatory cytokine that reduces pro-inflammatory cytokines such as IL-1β and TNF-α and inhibits leukocytes. In addition, NF-κB plays an important role in inflammatory signaling pathways and induces inflammatory cytokines and chemokines. *A. millefolium* extract down-regulated inflammation through this pathway.

#### 3.1.5 Ethanol

Ethanol has been shown to have significant adverse effects on the gastrointestinal system (GIS). It is known to cause irritation and inflammation in the GIS. In addition, prolonged ethanol consumption can lead to serious damage to the GIS, such as ulcers, bleeding, and even cancer [71]. Cavalcanti et al. [72] divided 18 rats into three groups and treated them with water (group 1), ranitidine (group 2), and *A. millefolium* extract (group 3). After 1 h, Cavalcanti induced gastric lesions with ethanol and indomethacin. The animals were then sacrificed to determine mucosal damage. Surprisingly, *A. millefolium* extract at the dose of 2000 mg/kg had better beneficial effects than ranitidine. Potrich et al. [43] investigated the gastroprotective activity of *A. millefolium*. They induced acute gastric lesions with ethanol and chronic gastric ulcers with acetic acid. Pretreatment with the plant extract reduced the lesion area. Furthermore, oral administration of the plant extract showed an increase in SOD and glutathione (GSH) concentration and a decrease in myeloperoxidase (MPO).

#### 3.1.6 Ethylene glycol

Ethylene glycol toxicity often results in the development of acute renal failure. The accumulation of calcium oxalate monohydrate (COM) crystals in renal tissue causes renal tubular necrosis, which leads to renal failure. At toxicologically significant concentrations, only COM crystals, and not oxalate, glycolaldehyde, or glyoxylate ions, caused necrotic cell death. The process by which COM crystals accumulate in the kidney involves adherence to the tubular cell membrane and subsequent internalization of the crystals, resulting in high concentrations in the organ. The metabolites have the ability to act as cytotoxins, resulting in central nervous system depression and cardiopulmonary and renal failure. In addition, glycolic acid can cause severe acidosis, while oxalate can precipitate as calcium oxalate in various tissues, including the kidneys [73]. Hassani et al. [44] investigated the preventive and medicinal properties of *A. millefolium* extract on ethylene glycol-induced nephrolithiasis in rats. The result showed a reduction in urinary oxalate concentration. It also increased the urinary citrate concentration. In addition, the result showed anti-inflammatory, diuretic, and antibacterial activities for the extraction of *A. millefolium*.

#### 3.1.7 Lipopolysaccharide (LPS)

Lipopolysaccharide (LPS) is a major component of the wall of Gram-negative bacteria and their endotoxin. As a mitogen, LPS induces cell proliferation in B lymphocytes and secretion of cytokines such as IL-1β and TNF-α in macrophages. LPS is heat-stable and has a long shelf life. These compounds have been known for many years to be the major factors in human septic shock. By binding to the CD14/TLR4/MD2 receptor complex, LPS causes the release of inflammatory cytokines, such as IL-1β and TNF-α, thus generating a very strong immune response in the mammalian body [74].

In a research study, a total of 19 compounds were detected in the essential oil of *A. millefolium* (AM-EO). The most abundant components of the oil were identified as artemisia ketones, accounting for 14.92 % of the total oil. Other compounds such as linalyl acetate, camphor, and 1,8-cineole included 11.51 %, 11.64 %, and 10.15 % of the total oil, respectively. The AM-EO showed that it could suppress the induced LPS-stimulated RAW 264.7 macrophage inflammatory responses, which include lowering in cellular nitric oxide (NO), production of superoxide anion, lipid peroxidation, and concentration levels of GSH. Such antioxidant activity is not due to an increase in levels of catalase (CAT), SOD, or glutathione peroxidase (GPx) activities. However, it could occur in line with down-regulation of inducible nitric oxide synthase (iNOS), COX-2, IL-6, TNF-α, and heme oxygenase-1 (HO-1) expression, which reduce its inflammatory response. Therefore, AM-EO can be used in many applications, including the treatment of inflammatory diseases in the future. COX-2 causes inflammation by converting arachidonic acid to prostaglandins. Also, iNOS activity leads to NO accumulation. AM-EO reduced the inflammatory response of macrophages by down-regulating iNOS, COX-2, TNF-α, and IL-6 [45].

Yaeesh et al. [75] investigated the survival rate and hepatoprotective properties of crude *A. millefolium* extract in d-galactosamine and lipopolysaccharide-induced hepatitis in mice. Pretreatment with *A. millefolium* reduced the mortality rate from 100 % to 40 %. It also reduced plasma liver enzyme concentration. These results show the hepatoprotective activity of the aqueous-methanol extract of *A. millefolium*. In an *in vitro* experiment, Chou et al. [45] investigated the antioxidant activities of AM-EO. Oxidative stress was induced in macrophages by LPS. AM-EO prevented lipid peroxidation, NO production, and GSH concentration. They concluded that this prevention is due to the reduction of COX-2, iNOS, TNF-α, and HO-1 expression. Thus, AM-EO can be used as an effective anti-inflammatory agent.

#### 3.1.8 Morphine

Morphine, a powerful narcotic painkiller, is widely used to relieve acute pain as well as to treat persistent severe pain. Morphine belongs to the group of alkaloids with a morphine framework and is found in the poppy plant. It has the ability to dissolve in water but not in lipids. Morphine-3-glucuronide and morphine-6-glucuronide are the main metabolites of morphine in the human body. Morphine metabolism takes place in the liver, kidneys, and brain. Most glucuronides are excreted in bile and urine. They are also considered highly polar metabolites that cannot cross the blood-brain barrier. Chronic exposure to opioids can decrease hippocampal morphine, which is a potent opioid analgesic used extensively for acute and long-term treatment of severe pain [76]. Mozafari et al. [46] investigated the neuroprotective properties of the aqueous extract of A*. millefolium* in morphine-induced rats. After morphine administration, the aqueous extract was administered orally at three different doses (100, 250, and 500 mg/kg). After 28 days, the activities of SOD and GPx were increased. The concentration of B cell lymphoma 2 (Bcl-2) was also significantly increased. However, the levels of caspase-3, MDA, and Bcl-2-associated X protein (Bax) were decreased. Morphine was found to increase caspase-3 and Bax expression and decrease Bcl-2 expression in the hippocampus. *A. millefolium* extract downregulated Bax and caspase-3 and upregulated Bcl-2, which resulted in inhibition of ischemia.

#### 3.1.9 Sodium lauryl sulfate (SLS)

Sodium lauryl sulfate (SLS) is an anionic surfactant used as an emulsifier in various pharmaceutical drug delivery systems, foaming dentifrices, cosmetic delivery systems, and even in the food industry [77]. Sodium lauryl sulfate irritation has been found in various tissues, including the respiratory mucosa [78]. The oral toxicity and tolerability of SLS, with and without other excipients, have been studied in dogs and rats by gavage, feed, or water [[79], [80], [81]]. According to the study by Tadić et al. [47], the oil extract of *A. millefolium* has anti-inflammatory properties. They artificially irritated the skin of 23 volunteers with SLS and tested the beneficial effect of the oil extract on them. Treatment with *A. millefolium* resulted in an increase in skin hydration of the irritated skin. Skin irritation caused an increase in skin pH. Treatment with oil extract caused the pH to return to normal.

#### 3.1.10 Streptozotocin (STZ)

Streptozotocin (STZ) belongs to the glucosamine-nitrosourea family of drugs that strongly suppress insulin production by pancreatic beta cells in mammals; it is used to treat certain cancers of the islets of Langerhans. STZ is used in medical research to develop a DM model [82,83]. Because of its toxic effects on pancreatic β-cells via DNA damage and stimulation of the inflammatory response, STZ is widely used to induce hyperglycemia in experimental animals [84].

When the hydroalcoholic extract of *A. millefolium* was injected into STZ-induced diabetic rats by Zolghadri at the dose of 100 mg/kg for 14 days, body weight and serum insulin level were increased. Glucose level, iNOS, and IL-1β gene expression were also reduced. These results demonstrate the protective effects of *A. millefolium* against DM [48].

According to the study of Karimi et al. [85] on *A. millefolium*, they demonstrated antioxidant and antidiabetic activities against STZ-induced diabetic rats. The hydroalcoholic extract of the plant was administered at a dose of 250 mg/kg for 21 days. After treatment, the activities of SOD and GPx were increased. However, MDA and blood glucose were reduced in the treatment group compared to the diabetic group. In DM, increased expression of Bax protein leads to renal tissue damage. In the study, plant administration reduced Bax expression and upregulated Bcl-2 expression.

An investigation was conducted on *A. millefolium* extract, which is known as a traditional compound with a hypoglycemic effect. They also investigated its effect on IL-1β and iNOS gene expression in pancreatic tissue of STZ-induced diabetic rats. Four groups of forty adult male rats were formed. For two weeks, *A.* millefolium extract was administered to the mice by injection (100 mg/kg/day). The diabetic rats showed an increase in the mRNA expression levels of IL-1β and iNOS genes. However, the extract-treated mice had higher insulin levels along with lower glucose levels and higher body weight compared to the diabetic control group. Therefore, it can be assumed that the reduction of IL-1β and iNOS gene expression, which has a protective effect on β-cells, is responsible for the beneficial effect of *A. millefolium* on STZ-induced DM. IL-1β activates the NF-κB pathway and leads to inflammation. The plant extract reduced inflammation by down-regulating the expression of IL-1β, iNOS, and COX-2 [48].

*A. millefolium* is a multi-faceted herb with notable health benefits, evidenced by its significant anti-hyperglycemic and anti-hyperlipidemic effects, as shown in studies where its extract led to increased body weight and decreased levels of serum triglycerides, LDL, and VLDL in rats. It exhibits hepatoprotective properties by preventing liver damage from CCl_4_ exposure, evidenced by reduced liver enzyme levels and absent histological changes in treated animals. Furthermore, *A. millefolium* demonstrates potent anti-inflammatory actions, decreasing levels of oxidative stress markers like malondialdehyde while boosting antioxidant defenses through increased superoxide dismutase and glutathione levels. The extract effectively modulates inflammatory pathways by downregulating crucial cytokines, such as IL-1β and TNF-α, and enzymes like COX-2 and iNOS. It protects gastric tissues from lesions caused by ethanol and acetic acid, aids renal health by reducing urinary oxalate and increasing citrate, and enhances skin hydration and pH balance in irritated skin. Additionally, it shows promise in diabetes management by normalizing blood glucose levels and improving insulin secretion while preserving pancreatic beta-cell function. The mechanisms of action underlying these effects include enhanced antioxidant activity, reduced inflammation, and improved cell survival signaling through the modulation of pro-apoptotic and anti-apoptotic proteins, making *A. millefolium* a promising candidate for therapeutic applications in metabolic disorders and inflammation-related conditions.

### 3.2 Achillea wilhelmsii

*A. wilhelmsii* C. Koch is usually found in different regions of Iran and is widely used in traditional Iranian medicine to treat symptoms of gastrointestinal and cardiovascular diseases [[86], [87], [88]]. This plant contains sesquiterpene lactones and flavonoids, which are effective in lowering blood lipids and hypertension. The aerial parts of the plant, such as flowers, leaves, twigs, and fruits, are used for therapeutic applications [18]. The following are the flavonoids found in *A. wilhelmsii*; 5-demethylsinensetin, isoorientin, isoschaftoside, isovitexin, salvigenin, apigenin, luteolin, quercetin, rutin, artemitin, isoschaftoside, isovitexin, penduletin, salvigenin, santoflavone, and vitexin [89]. The protective effects of *A. wilhelmsii* against natural and chemical toxins are shown in Table 2.

**Table 2:** Protective effects of *A. wilhelmsii* against natural and chemical toxins.

Toxin/Noxious	Model	Extract/Essential Oil/Dose/Concentration	Results	References
Acetaminophen	Male Wistar rats	Essential oil (100, and 200 mg/kg)	↓ Activity of CYP450 (p < 0.05)	[49]
			↓ Activity of ALT and AST (p < 0.05)	
			↑ GSH (p < 0.05)	
			↑ GST activity (p < 0.05)	
Acetaminophen	Male Wistar rats	Essential oil (100, and 200 mg/kg)	↓ SOD activity (p < 0.05)	[98]
			↓ MDA (p < 0.05)	
			↑ GSH (p < 0.05)	
Acetic acid	Male Wister rats	Extract; 6.25, 12.5, 25, 50, and 100 mg/kg/d	↓ Macroscopic and microscopic scores of colitis (p < 0.05)	[50]
Acetic acid	Rats	Hydroalcoholic extract (50, 100, 200 mg/kg)	↓ IL-6 (p = unknown)	[100]
			↓ TNF-α (p = unknown)	
			↓ MPO (p = unknown)	
Alloxan	Mice	Water, hydroalcoholic, and ethyl acetate extract for 14 days	↓ FBG (p < 0.05)	[101]
			↓ TNF-α (p < 0.001)	
HPV	HeLa cervical cancer cell and LIN28B and p53	0–200 μg/ml for 24–72 h.	↓ HeLa cells growth (p < 0.05)	[162]
			↓ LIN28B mRNA (p < 0.05)	
			↑ p53 mRNA (p < 0.05)	
LPS	Male BALB/c mice	Ethanolic extract (150–300 mg/kg for 24 h)	↓ BALF (p = unknown)	[104]
			↓ TNF-α (p = unknown)	
			↓ Induced pathological alterations (p = unknown)	
			↑ Weight (p = unknown)	
Pentylenetetrazole	Rats	Hydroalcoholic extract (100, 200, and 400 mg/kg)	↓ MDA (p < 0.001)	[51]
			↑ GTCS (p < 0.001)	
			↑ Total thiol concentration (p < 0.01)	

#### 3.2.1 Acetaminophen

Acetaminophen is a common antipyretic with hepatotoxicity above therapeutic blood levels [90]. Because of the increased use of combination therapy with acetaminophen in over-the-counter cold medicines or prescription pain relievers, the determination of acetaminophen toxicity has recently become more complex [[91], [92], [93]]. Many studies have investigated the mechanism of toxicity associated with ingestion of high doses of acetaminophen. It was shown that in the metabolic process through the CYP450 enzyme in conversion to N-acetyl-*p*-benzoquinone imine (NAPQI), which could be reduced by glutathione, at the toxic dose, the glutathione storage in the body became low and insufficient for the reduction process of NAPQI. NAPQI could bind to liver cells and cause liver necrosis [[94], [95], [96], [97]]. According to an *in vivo* study conducted by Dadkhah et al. [49] in 2014, the essential oil of *A. wilhelmsii* at the dose of 100 and 200 mg/kg has a protective effect against acetaminophen-induced liver damage. The results showed that injection of plant essential oil increased glutathione s-transferase (GST) activity and concentration compared to the negative control group. It also decreased hepatic enzyme activities such as alanine transaminase (ALT) and aspartate aminotransferase (AST). In another *in vivo* study, they showed that the essential oil of *A. wilhelmsii* also reduced the activity of CAT and SOD. In addition, the increase in glutathione (GSH) concentration and the decrease in MDA levels were other effects associated with the use of the plant essential oil [98].

#### 3.2.2 Acetic acid

Acetic acid, which has antibacterial and antifungal properties, is a synthetic carboxylic acid. Although its mechanism of action is not fully understood, undissociated acetic acid has the potential to increase lipid solubility, which may lead to increased accumulation of fatty acids on the cell membrane or other cell wall structures. Acetic acid can interfere with the process of carbohydrate metabolism, leading to the death of the organism. The acetyl group derived from acetic acid serves as a crucial element in the biochemistry of almost all living organisms. However, the concentration of free acetate in cells is carefully regulated at low levels to prevent interference with the pH control of cell contents. Certain bacteria, including the genus Acetobacter and Clostridium acetobutylicum, are used to produce and excrete acetic acid. These bacteria are commonly found in water, food, and soil. In addition, acetic acid is a natural byproduct of fruit and other food spoilage. It is one of the common ingredients in vaginal lubricants in humans and other primates, where it appears to act as a mild antibacterial agent [99].

In a study conducted by Ghobadi and Heydarian et al. [50], the therapeutic effect of *A. wilhelmsii* aqueous extract on acetic acid-induced ulcerative colitis in rats was evaluated. The results showed that *A. wilhelmsii* aqueous extract significantly reduced the severity of colitis. They concluded that *A. wilhelmsii* aqueous extract has the potential as a therapeutic agent for the treatment of ulcerative colitis. In another study, colitis was induced in rats by rectal administration of 4 % acetic acid. They were treated with hydroalcoholic extract of *A. wilhelmsii* at concentrations of 50, 100, and 200 mg/kg for 48 h. *A. wilhelmsii* improved the macroscopic and microscopic symptoms of induced colitis. The extract also reduced pro-inflammatory mediators, including IL-6, TNF-α, and MPO in colonic tissue. It also decreased mucosal toll-like receptor 4 (TLR-4) expression with a significant decrease in TNF-α and IL-6 production [100].

#### 3.2.3 Alloxan

Khazneh et al. [101] investigated the hypoglycemic activity of *A. wilhelmsii* and its effect on inflammatory mediators. Oral treatment of alloxan-induced mice with different fractions of the plant for 20 days was associated with a reduction in blood glucose.

#### 3.2.4 Toxic and heavy metals

In recent years, the expansion and development of various industries, in conjunction with the rise in environmental pollutants, have led to a marked increase in the significance of heavy metals. These metals, most notably cadmium, lead, mercury, and arsenic, contribute to air, water, and soil contamination. Humans are exposed to these metals through inhalation, ingestion, and dermal absorption, which can have detrimental health consequences. Research indicates that these metals can harm humans by impairing brain function and damaging various organs, including the brain, kidneys, lungs, and liver. Prolonged exposure to these metals can result in neurological and muscular damage, as well as diseases such as amyotrophic lateral sclerosis, Alzheimer's disease, Parkinson's disease, and cancer [102].

Phytoremediation has emerged as a viable, sustainable method for mitigating heavy metal pollution. Various studies have demonstrated the effectiveness of various plant species in absorbing and accumulating heavy metals from contaminated soils and air. This approach not only contributes to the remediation of polluted environments but also enhances ecological safety for living organisms by monitoring transportation emissions and their impact on ecosystems. In recent years, researchers have focused on specific plant species with high bioconcentration and bioaccumulation potential. The study of *A. wilhelmsii* and *Cardaria draba* along suburban roads in Hamadan provides insight into their feasibility for the removal of heavy metal (Zn, Pb, Ni). Previous research has shown that species with significant bioaccumulation factors (BAF) and bioconcentration factors (BCF) are essential for successful phytoremediation efforts. The methodology used to assess heavy metal concentrations, including the use of inductively coupled plasma optical emission spectroscopy, is well documented in the literature. Acid digestion of plant and soil samples is standard practice to prepare them for accurate heavy metal analysis. The results of the study indicate mean BCF and BAF values greater than 1 for both *A. wilhelmsii* and *C. draba*, indicating a significant capacity for metal uptake. In particular, transfer factors greater than 1 for zinc in *A. wilhelmsii* and for zinc and lead in *C. draba* reflect their effectiveness in phytoremediation. Similarly, high metal accumulation index values in various plant parts underscore their role in potential bioremediation strategies. The results support the argument that *A. wilhelmsii* and *C. draba* can be effectively used for biomonitoring and bioremediation in areas affected by heavy metal pollution [103].

#### 3.2.5 Lipopolysaccharide (LPS)

The anti-inflammatory activity of ethanolic extract of *A. wilhelmsii* on acute lung injury (ALI) was also investigated. Honari et al. [104] divided LPS-induced ALI mice into four groups; the first group was treated with saline, the second group was treated with dexamethasone, and the third and fourth groups were treated with two different doses of *A. wilhelmsii*. Twenty-four hours after LPS administration, TNF-α was decreased in the third and fourth groups compared to the control group.

#### 3.2.6 Pentylenetetrazol (PTZ)

Pentylenetetrazol (PTZ) has been one of the most commonly used convulsive chemical stimulants for the past 20 years. One of the models that causes epilepsy is the PTZ-reactivated chronic seizure [105]. PTZ has been shown to induce epileptiform activity in hippocampal pyramidal neurons, characterized by bursts of sodium-dependent action potentials [106]. When PTZ-induced seizure rats were treated with *A. wilhelmsii* hydroalcoholic extract (100, 200, and 400 mg/kg) prior to PTZ injection by Hosseini et al. [51], a reduction in MDA levels was observed. Pretreatment with the plant extract also resulted in an increase in total thiol concentration.

*A. wilhelmsii* was shown to possess various protective and therapeutic effects, particularly against liver and gastrointestinal damage. The essential oil of *A. wilhelmsii* offered protection against liver damage by increasing GST activity and concentration while simultaneously decreasing hepatic enzyme levels of ALT and AST. The essential oil also elevated GSH levels and reduced MDA concentrations. The aqueous extract of *A. wilhelmsii* significantly mitigated the severity of ulcerative colitis in rats, improving both macroscopic and microscopic symptoms while decreasing pro-inflammatory markers such as IL-6 and TNF-α, and lowering the expression of mucosal TLR-4. Furthermore, researchers demonstrated the hypoglycemic activity of *A. wilhelmsii* in alloxan-induced diabetic mice, resulting in significant reductions in blood glucose levels. In terms of phytoremediation, *A. wilhelmsii* exhibited a high capacity for metal uptake, indicating their potential for biomonitoring and bioremediation in heavy metal-contaminated environments. Additionally, *A. wilhelmsii* demonstrated anti-inflammatory effects in models of acute lung injury by reducing TNF-α levels compared to control groups. Lastly, in trials involving seizures, treatment with hydroalcoholic extracts of *A. wilhelmsii* resulted in reduced MDA levels and increased total thiol concentrations, suggesting neuroprotective effects against oxidative stress in seizure conditions. Overall, the various extracts of Achillea wilhelmsii show significant potential for therapeutic applications in liver protection, gastrointestinal health, glucose regulation, inflammation reduction, and neuroprotection.

### 3.3 Achillea fragrantissima

*A. fragrantissima* is a traditional herbal medicine used by people in the Middle East. This traditional herbal medicine is used to treat a variety of ailments such as respiratory diseases, eye infections, smallpox, fever, gastrointestinal disorders, dysmenorrhea, headache, fatigue, and DM [8,[107], [108], [109]]. It also has insecticidal, antiviral, antimicrobial, and antioxidant activities [[110], [111], [112]]. These properties relate to its phytochemicals such as; acerosin, cirsimaritin, cirsiliol, luteolin, apigenin, caffeic acid, santolina triene, α-thujene, α-pinene, α-fenchene, camphene, benzaldehyde, sabinene, β-pinene, 2,3-dehydro-1,8-cineol, yomogi alcohol, α-terpinene, *p*-cymene, limonene, santolina alcohol, β-phellandrene, 1,8-cineole, γ-terpinene, linalool, α-thujone, β-thujone, myrcenol, fenchol, chrysanthenone, α-terpineol, methyl chavicol, verbenone, carvone, isobornyl acetate, thymol and carvacrol [55]. The protective effects of *A. fragrantissima* against natural and chemical toxins are shown in Table 3.

**Table 3:** Protective effects of *A. fragrantissima* against natural and chemical toxins.

Toxin/Noxious	Model	Extract/Essential Oil/Dose/Concentration	Results	References
Adriamycin	Rats	Extract (400 and 800 mg/kg for 14 days)	↓ IL-1β (p ≤ 0.05)	[52]
			↓ TNF-α (p ≤ 0.05)	
			↓ CK-MB activity (p ≤ 0.05)	
			↓ LDH activity (p ≤ 0.05)	
			↓ TBARS (p ≤ 0.05)	
			↑ GPx (p ≤ 0.05)	
			↑ GSH (p ≤ 0.05)	
			↓ IL-6 (p ≤ 0.05)	
*Trypanosoma evansi*	Wister albino mature male rats	Methanolic extract (1000, and 500 mg/kg)	↑ PCV (p ≤ 0.05)	[163]
			↑ Hb concentration (p ≤ 0.05)	
			↑ TLC (p ≤ 0.05)	
			↑ LC (p ≤ 0.05)	
			↑ GSH (p ≤ 0.05)	
			↓ MDA (p ≤ 0.05)	
β-amyloid	Neuro2a cells	TTF extract (3, 14, and 32 nM)	↓ ROS (p = unknown)	[164]
			↓ SAPK/JNK (p < 0.001)	
			↓ ERK 1/2 (p < 0.001)	
β-amyloid	Neuro2a cells	Achillolide A extract (32, 160, and 320 nM)	↑ viability (p < 0.001)	[165]
			↓ ROS (p < 0.001)	
			↓ SAPK/JNK (p < 0.001)	

#### 3.3.1 Adriamycin

Adriamycin (Adr) is widely recognized as one of the most potent chemotherapeutic agents available and is used extensively in the treatment of various tumors. The multiple toxicities associated with Adr hinder its clinical use, including cardiac, renal, and pulmonary toxicities. Administration of Adr may be associated with acute cardiac toxicity ranging from ventricular and atrial arrhythmias to congestive heart failure. Studies indicate that the cardiotoxicity of Adr is due to the production of free radicals and ROS, which could damage the cell membrane lipid and release lipid peroxide and its derivatives, followed by membrane lipid damage. In addition, there is increasing evidence that Adr could induce inflammatory effects in the myocardium and vasculature, subsequently producing several pro-inflammatory mediators such as TNF-α. The IL-1β is known as an initiator cytokine that has an excellent function to regulate inflammatory pathways. It has been shown that IL-1β contributes to the Adr-induced increase in IL-6 levels and plays an important role in Adr-induced cardiotoxicity [113]. Hijazi et al. [52] evaluated the antioxidant activity of *A. fragrantissima* against Adr-induced cardiotoxicity in rats. They administered the plant extract orally at two doses (400 and 800 mg/kg) as pretreatment after two weeks. The results showed a reduction in TNF-α and IL-1β. It also increased GPx and GSH concentrations.

#### 3.3.2 Carrageenan

Induction of paw edema by carrageenan is a common technique used to evaluate anti-inflammatory efficacy [114]. It is a useful technique for nonsteroidal anti-inflammatory drugs (NSAIDs) and has long been used to study new NSAIDs [115]. The technique is useful for studying new NSAIDs and is highly sensitive [116]. Injection of carrageenan leads to the development of edema, which triggers an acute and localized inflammatory response. During the initial phase (0–1h), the first mediators produced are serotonin, histamine, and bradykinin. The second phase involves the release of prostaglandins and several cytokines such as IL-6, IL-1β, IL-10, and TNF-α [53]. Abdel-Rahman et al. [117] demonstrated the anti-inflammatory and anti-ulcerogenic properties of dichloromethane extract of *A. fragrantissima* by oral administration of 200 and 400 mg/kg to carrageenan-induced rats. The results showed a reduction in the edema rate. Also, pretreatment of pylorus ligation-induced gastric ulceration in rats with plant extracts reduced gastric secretions.

*A. fragrantissima* exhibits several mechanisms of action that highlight its protective and therapeutic effects, particularly against cardiotoxicity and inflammation. Administration of *A. fragrantissima* extract significantly reduced levels of pro-inflammatory cytokines TNF-α and IL-1β, which are involved in cardiotoxicity. The extract also increased the concentrations of GSH and GPx, enhancing the antioxidant defense mechanisms and reducing oxidative stress. Furthermore, *A. fragrantissima* effectively decreased edema formation, indicating its ability to inhibit the early inflammatory mediators such as serotonin, histamine, and bradykinin, as well as the release of prostaglandins and cytokines. Additionally, the dichloromethane extract of the plant has shown anti-ulcerogenic effects by reducing gastric secretions in pylorus ligation-induced gastric ulceration, suggesting multiple therapeutic uses of *A. fragrantissima* through its antioxidant, anti-inflammatory, and gastroprotective actions. Overall, the mechanisms include enhancing antioxidant enzyme activity, reducing inflammatory cytokine levels, and protecting epithelial tissues, making *A. fragrantissima* a promising candidate for mitigating Adr-related toxicities and inflammatory conditions.

### 3.4 Achillea santolina

*A. santolina* is a plant that grows in barley and fallow fields [118]. The compounds, flavones and polyphenols of *A. santolina* have antimicrobial and anti-inflammatory activities [119,120]. Due to its depurative, antispasmodic, and carminative activities, *A. santolina* is used in traditional medicine for the treatment of gastrointestinal disorders [89]. Furthermore, A. santolina is used to control hypoglycemia and has been approved as an alternative therapy for DM; this is due to the abundance of several phenolic compounds that have been shown to have hypoglycemic activity *in vitro* and *in vivo* [121,122]. Apigenin, luteolin, lutein, 7-*O*-β-*d*-glucoside, and rutin are some of the major flavonoids found in *A. santolina* [89]. The protective effects of *A. santolina* against natural and chemical toxins are shown in Table 4.

**Table 4:** Protective effects of *A. santolina* against natural and chemical toxins.

Toxin/Noxious	Model	Extract/Essential Oil/Dose/Concentration	Results	References
*Leishmania infantum*	The standard strain of *L. Infantum*	10, 50, 100, 200, 500, and 1000 mg/ml	↓ Viability (p < 0.05)	[166]
Streptozotocin	Rats	Extract (0.1 g/kg/day for 30 days)	↑ SOD (p < 0.05)	[121]
			↑ CAT (p < 0.05)	
			↑ GSH (p < 0.05)	
			↑ Blood glucose level (p < 0.05)	
			↓ Serum NO (p < 0.05)	
			↓ MDA (p < 0.05)	
			↓ PCO (p < 0.05)	

#### 3.4.1 Aflatoxin

Aflatoxin, which is an extremely toxic mycotoxin, has the ability to withstand freezing and high-temperature conditions such as cooking. The fungi *Aspergillus flavus*, *Aspergillus nomiu,* and *Aspergillus parasiticus*, which grow in a wide range of temperatures, produce different types of aflatoxins (from 12 to 42C) [123]. Aflatoxin can be produced during both pre-harvest and post-harvest storage, which is one of the most challenging issues to control [124]. Humans are exposed to aflatoxins through the consumption of foods such as eggs, wheat, maize, milk, and dairy products [125]. It causes hepatotoxicity, nephrotoxicity, carcinogenicity, immunotoxicity, and mutagenicity in both acute and chronic situations [[126], [127], [128], [129]]. A study was conducted with the aim of evaluating the antifungal potential of *A. santolina* and *Calendula officinalis* essential oils and their compounds for cyclophosphamide-treated fungal infection. The result showed a significant reduction in the production of aflatoxin produced by *A. flavus* in a dose-dependent manner. The oil blend also showed antioxidant and radical scavenging activities [54].

#### 3.4.2 Streptozotocin

The anti-diabetic effects of *A. santolina* were evaluated in STZ-induced diabetic rats. Yazdanparast et al. [121] divided 22 rats into three groups. Group 3 was administered with plant extract daily for one month at the dose of 0.1 g/kg. Treatment with the plant extract reduced blood glucose and prevented weight loss during the study. It also has beneficial effects on the antioxidant system and on protein oxidation. Their results showed that *A. santolina* extract could reduce the levels of MDA and serum NO and increase the levels of GSH, CAT, and SOD.

### 3.5 Achillea biebersteinii

*A. biebersteinii* Afan. is a 20–50 cm long non-woody perennial aromatic herb. The plant usually grows in clusters. The leaves of this plant can grow up to 10 cm long [130]. Many biological activities of *A. biebersteinii* have been identified, including hypoglycemic, antioxidant, neuroprotective, anti-ulcer, anti-cancer, anti-inflammatory, wound healing, and antibacterial properties [[131], [132], [133], [134], [135], [136], [137], [138]]. *A. biebersteinii* is an ancient herb, stems are straight, simple or branched from the base; a yellow flowering plant. It's 30–60 cm tall with leaves up to 10 cm tall, and its flowering season is April-May [139,140]. *A. biebersteinii* has been used in traditional medicine for its wound-healing, antibacterial and antifungal properties. Scientific evidence has reported that as well as its antioxidant, anti-inflammatory, and antinociceptive activities [131,[141], [142], [143]]. Among the various flavonoids found in *A. biebersteinii*, the following are the most important ones: 5,7-dihydroxy-3,3′,4′-trimethoxy flavone, santin, quercetagetin 3,6,3′-trimethyl ether, quercetagetin 3,6-dimethyl ether, apigenin, luteolin, quercetin, rutin, axillarin, 3,8-dimethylherbacetin, jaceidin, and kaempferol [89]. The protective effects of *A. biebersteinii* against natural and chemical toxins are shown in Table 5.

**Table 5:** Protective effects of *A. biebersteinii* against natural and chemical toxins.

Toxin/Noxious	Model	Extract/Essential Oil/Dose/Concentration	Results	References
Carbon tetrachloride	Rats	Essential oil (0.2 ml/kg)	↓ SGOT (p < 0.001)	[167]
			↓ SGPT (p < 0.01)	
			↓ GGT (p < 0.001)	
			↓ ALP (p < 0.01)	
			↓ Bilirubin (p < 0.001)	
			↓ Cholesterol (p < 0.001)	
			↓ TG (p < 0.001)	
			↓ VLDL (p < 0.001)	
			↑ HDL (p < 0.01)	
			↑ LDL (p < 0.05)	
Dimethoate	Pigs	Aqueous extract (50 and 100 mg/kg for 2 weeks)	↓ AST (p = unknown)	[168]
			↓ ALT (p = unknown)	
			↓ ALP (p = unknown)	
			↓ Lesions (p = unknown)	
Ethanol	Rats	Aerial parts extract (200 mg/kg for 1 week)	↓ GSH (p < 0.0001)	[144]
			↓ MDA (p < 0.0001)	
			↓ SOD (p < 0.0001)	
			↓ Gastric volume (p < 0.0001)	
			↓ Total acidity (p < 0.0001)	
			↓ Lesions count (p < 0.0001)	

#### 3.5.1 Ethanol

Abd-Alla et al. [144] conducted a study to evaluate the protective effect of *A. biebersteinii* against ethanol-induced gastric ulcers. The plant ethyl acetate extract (EAE) was administered orally to rats for seven days. After one week of treatment with EAE, gastric volume, total acidity, and number of lesions were reduced compared to the non-treated group. EAE also had antioxidant activity. Treatment with the extract reduced GSH, MDA, and SOD concentrations even better than the ranitidine group (positive control group).

### 3.6 Achillea odorata

*A. odorata* is widely used as a medicinal plant in Algerian folk medicine. Its potential antinociceptive, antioxidant, and anti-inflammatory compounds could be assumed as drug candidates [145]. The chemical composition of the essential oil of *A. odorata* is listed below:

α-pinene, camphene, sabinene, β-pinene, limonene, 1,8-cineol, γ-terpinene, *trans*-sabinene hydrate, α-thujone, β-thujone, chrysanthenone, α-campholenal, camphor, *trans*-pinocarveol, *trans*-verbenol, pinocarvone, borneol, terpinen-4-ol, myrtenal, α-terpineol, myrtenol, *trans*-chrysanthenyl acetate, piperitone, iso-thymol, bornyl acetate, myrtenyl acetate [146].

#### 3.6.1 Carrageenan

Boutennoun et al. [145] investigated the antinociceptive and anti-inflammatory effects of *A. odorata* in mice. Oral pretreatment with *A. odorata* extract at the dose of 200, 400, and 600 mg/kg on acetic acid-induced algesia mice caused a reduction in the number of lesions. Pretreatment with the plant extract in carrageenan-induced paw edema also resulted in a reduction in paw thickness and MDA levels and an increase in GSH, CAT, and SOD levels.

The protective effects of other *Achillea* species against natural and chemical toxins are shown in Table 6.

**Table 6:** Protective effects of other species of *Achillea* against natural and chemical toxins.

Toxin/Noxious	Species	Model	Extract/Essential Oil/Dose/Concentration	Results	References
12-0-tetradecanoylphorbol acetate	*A. ageratum*	Swiss mice	Chloroform extract (1,3, and 5 mg/ear)	↓ Acute edema (p < 0.001)	[169]
				↓ Chronic edema (p < 0.0001)	
				↓ MPO activity (p < 0.001)	
Acetic acid, and carrageenan	*A. odorata*	Mice	Extract (200, 400, and 600 mg/kg)	↑ antinociceptive activity (p < 0.001)	[145]
				↓ Paw thickness (p < 0.001)	
				↓ MDA (p < 0.001)	
				↑ GSH (p < 0.001)	
				↑ CAT activity (p < 0.001)	
				↑ SOD activity (p < 0.001)	
Hydrogen peroxide	*A. falcata*, *A. crithmifolia*, *A. nobilis*, *A. millefolium*, and *A. teretifolia*	Human erythrocyte and leucocyte	*Achillea* extract (unknown dose)	↓ CAT (p = unknown)	[170]
				↓ SOD (p = unknown)	
				↓ GPx (p = unknown)	
				↑ GSH (p = unknown)	
				↓ LPO (p = unknown)	
Hydrogen peroxide	*A. alpina*	H9c2 cell line	Achillinoside from *Achillea alpina* (25, 50, and 100 μg/ml)	↓ Caspase-3 (p < 0.01)	[171]
				↓ Caspase-9 (p < 0.01)	
				↑ Cell viability (p < 0.01)	
LPS	*A. acuminata*	Macrophage	Zaluzanin D (10, 25, and 50 μM)	↓ IL-6 (p < 0.01)	[172]
				↓ TNF-α (p < 0.01)	
				↓ IL-1β (p < 0.01)	
				↓ NO content (p < 0.01)	
				↓ iNOS (p < 0.01)	
				↓ COX-2 (p < 0.01)	
				↓ P65 (p < 0.01)	
				↑ iκbα (p < 0.05)	
				↓ MPO activity (p < 0.01)	
				↓ MDA (p < 0.01)	
				↑ SOD (p < 0.01)	
				↓ NF-κB (p < 0.01)	
Surgery	*A. cretica*	Rat	Achillea cretica extract (100, 200, and 400 mg/kg for 28 days)	↓ TNF-α (p < 0.005)	[173]
				↓ VEGF (p < 0.05)	
				↓ IL-6 (p < 0.01)	

## 4 Future prospects

The growing body of evidence on *Achillea* spp., particularly *A. millefolium*, highlights their complex potential in attenuating toxicities and addressing health challenges. These findings presents *Achillea* spp. as a potential candidates for pharmaceutical agents targeting various diseases. Also, clinical and preclinical data validate their therapeutic promise. For instance, hydroalcoholic extracts of *A. millefolium* induced apoptosis in human gastric cancer cells via dose- and time-dependent mechanisms and its antidiabetic potential, evidenced by reduced blood glucose and lipid levels in diabetic rat models, aligns with traditional uses for metabolic disorders [147]. However, research on *Achillea* species in toxicity studies has several challenges. Firstly, different extraction methods and using various methods and solvents lead to different outcomes in toxicity research [148]. Also, various exposure durations cause differences in results. These several results make it hard to standardize a protocol and design a clinical trial. Furthermore, bioactivity variability across *Achillea* species adds complexity [149]. Another clinical issue is an assessment of the risk-benefit of a therapeutic dose of *Achillea* species [150,151]. Also, it seems that plant extract shows its effects in poly-pharmacological pathways and it is difficult to clarify its safety. To overcome these problems, tiered solvent testing can be performed to characterize extraction methods and standardize safety and bioactivity. Also, techniques such as molecular docking can be helpful to isolate bioactive compounds rather than crude extracts [152]. Combining the traditional knowledge and pharmacological regulatory rules, *Achillea* spp. could be utilized against different diseases.

## 5 Conclusion

**Fig. 3:**
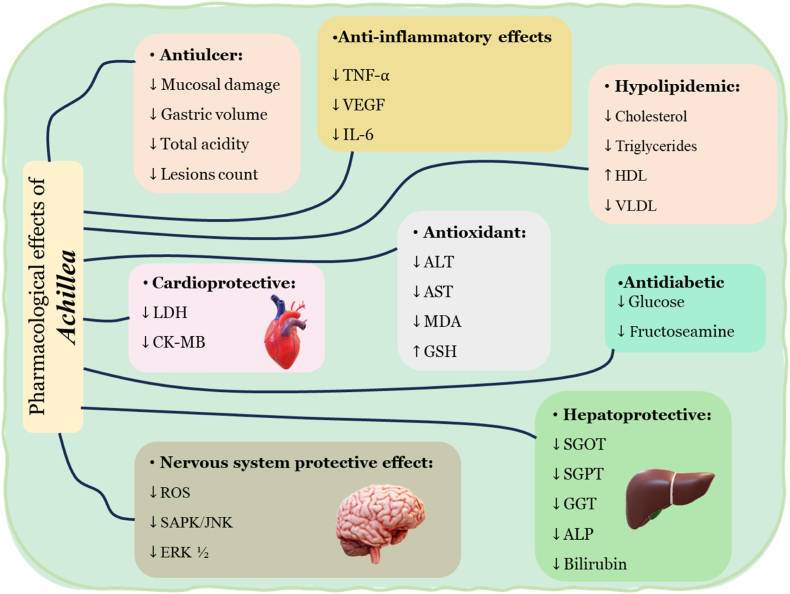
Pharmacological effects of *Achillea* species.

The *Achillea* genus, which belongs to the Asteraceae family, comprises a number of species that have been demonstrated to possess therapeutic properties. These species include *A. millefolium*, *A. wilhelmsii*, *A. fragrantissima*, *A. santolina*, *A. biebersteinii*, *A. ageratum*, *A. odorata*, *A. cretica*, and *A. nobilis*. In the contemporary context of increased exposure to natural and chemical toxins due to industrial development, the protective effects of *Achillea* against such toxins have significant implications for medical science. This review systematically evaluates the protective effects of various *Achillea* species through a comprehensive analysis of *in vivo* and *in vitro* studies, as summarized in Fig. 3. *A. millefolium* has been shown to possess noteworthy anti-hyperglycemic and anti-hyperlipidemic properties while concurrently enhancing gastroprotective activity and offering protection against ocular toxicity. Additionally, it has been observed to reduce inflammatory responses and augment antioxidant indices. *A. wilhelmsii* has been shown to be effective against hepatic damage and colitis, reducing pro-inflammatory measures and enhancing total thiol concentrations. *A. fragrantissima* has been shown to exhibit cardioprotective effects by decreasing inflammation and promoting antioxidant activity. The review also discusses the protective effects of other *Achillea* species against various toxins. Collectively, these findings suggest that *Achillea* species could serve as potential ameliorative agents against toxicity; however, further clinical trials are necessary to establish their efficacy in both animal models and human applications.

## CRediT authorship contribution statement

**Mohammad Mahdi Dabbaghi:** Writing – review & editing, Writing – original draft, Supervision, Methodology, Investigation. **Mohammad Saleh Fadaei:** Writing – review & editing, Writing – original draft, Methodology, Investigation. **Maral Goldoozian:** Writing – review & editing, Writing – original draft, Methodology, Investigation. **Mohammad Reza Fadaei:** Writing – review & editing, Writing – original draft, Supervision. **Vafa Baradaran Rahimi:** Writing – review & editing, Writing – original draft, Investigation, Conceptualization. **Vahid Reza Askari:** Writing – review & editing, Writing – original draft, Visualization, Validation, Supervision, Methodology, Investigation, Conceptualization.

## Ethics Approval and consent to participate

Not applicable.

## Availability of data and materials

No data was used to support the findings of this study.

## Human and animal rights

No animals/humans were used for studies that are the basis of this research.

## Funding

Declared none.

## Declaration of competing interest

The authors declare that they have no known competing financial interests or personal relationships that could have appeared to influence the work reported in this paper.

## Data Availability

No data was used for the research described in the article.
